# Healing chronic idiopathic eosinophilic pneumonia using mepolizumab alone without corticosteroids

**DOI:** 10.1007/s00508-024-02406-5

**Published:** 2024-08-08

**Authors:** Katharina Moritz, Carolina Amelunxen, Michael Bergmann, Florian Vafai-Tabrizi, Georg-Christian Funk

**Affiliations:** Medical Department II with Pneumology and Karl-Landsteiner Institute for Pneumology and Pulmonary Oncology, Klinik Ottakring, Montleartstraße 37, Pavillon 26 | Ebene B, 1160 Vienna, Austria

**Keywords:** Anti-IL‑5 monoclonal antibody, Eosinophilic pneumonia, Mepolizumab, Interstitial lung disease, Pulmonary inflammation

## Abstract

While glucocorticoids remain the standard first-line treatment for chronic idiopathic eosinophilic pneumonia (CIEP), the long-term use is marred by significant side effects. This case study explores the effectiveness of mepolizumab, an anti-interleukin‑5 (IL-5) monoclonal antibody, as a novel corticosteroid-free alternative in treating CIEP. A 50-year-old woman presented with a 3-week history of progressive shortness of breath, dry cough and night sweats. The blood tests showed eosinophilia, and chest radiography identified lung consolidations. The CIEP was confirmed, ruling out other conditions through a detailed clinical and bronchoscopic work-up. The patient declined to be treated with systemic glucocorticoids. Treatment with mepolizumab was remarkable for effectively resolving symptoms and improving radiological findings without any prior or concurrent glucocorticoid therapy. Notably, the patient remained relapse-free over a 2-year follow-up, underscoring mepolizumab’s efficacy as a corticosteroid-free treatment for CIEP. This case study calls for further research into anti-IL‑5 treatment of rare respiratory conditions.

## Introduction

Chronic idiopathic eosinophilic pneumonia (CIEP) is a rare eosinophilic lung disease marked by persistent respiratory symptoms and bilateral pulmonary infiltrates [1]. Predominantly affecting middle-aged women, the symptoms include dyspnea, cough, and fever [2]. The elusive nature of the etiology of CIEP, marked by a complex immune response, further affirms its idiopathic classification [3, 4].

While systemic glucocorticoids are central to CIEP treatment, their prolonged use not only leads to risks, such as osteoporosis and hypertension but also over half of the patients relapse upon dose reduction or cessation. Furthermore, one third of these patients experience multiple relapses, necessitating extended treatment, highlighting the need for safer alternatives [5]. Anti-interleukin‑5 (IL-5) plays a crucial role in eosinophil activity, and mepolizumab, an antibody targeting IL‑5, has been effective in treating severe eosinophilic asthma [6–8]. This report details a unique CIEP case managed with mepolizumab without glucocorticoids, underscoring its potential as a viable alternative in CIEP treatment.

## Case report

A 50-year-old female with an active smoking habit was referred to our pneumological outpatient clinic. She presented with a 3-week history of progressive shortness of breath, dry cough, and night sweats without a notable medical history or recent medication changes. Laboratory tests showed significant peripheral eosinophilia (1.68 G/L i.e. 25%), normal blood leukocyte count, slightly elevated C‑reactive protein (CRP), and negative myeloperoxidase anti-neutrophil cytoplasmic antibody (ANCA) and proteinase 3‑ANCA.

**Fig. 1:**
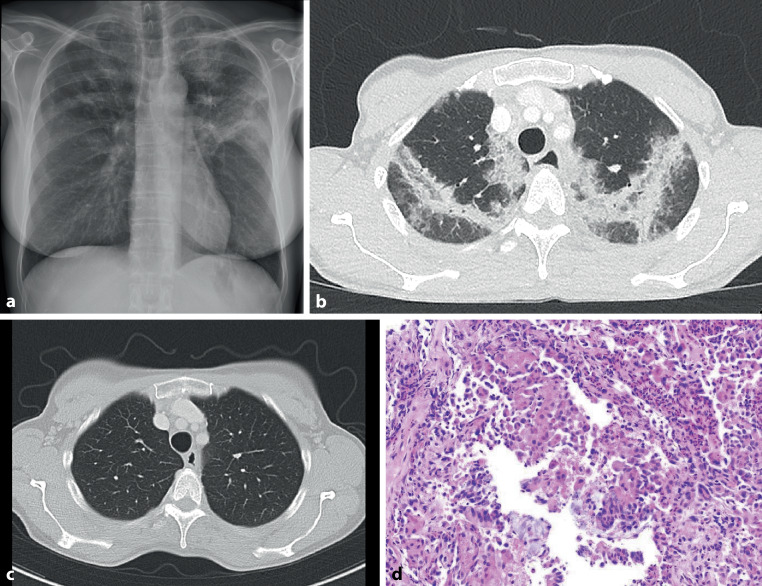
**a** Chest X‑ray prior to treatment depicting extensive upper lobe consolidations (posteroanterior view). **b** Chest computed tomography (CT) scan before treatment (transverse plane, upper lobes), illustrating similar extensive upper lobe consolidations, consistent with the X‑ray findings. **c** Chest CT scan 9 months post-treatment, displaying complete resolution of the eosinophilic pneumonia (transverse plane, upper lobes). **d** Transbronchial lung biopsy photomicrograph (× 200 magnification H&E stain) showing dense intra-alveolar and interstitial eosinophilic infiltration

Figure 1 illustrates the relevant imaging and pathological results. The chest X‑ray (Fig. 1a) revealed bilateral upper lung consolidations with a peripheral emphasis, and the chest computed tomography (CT) confirmed bilateral upper lobe consolidation (Fig. 1b, c). Spirometry, body plethysmography, diffusing capacity of the lungs for carbon monoxide (DLCO) and arterial blood gas were normal; however, a markedly elevated fractional exhaled nitric oxide (FENO) at 130 ppm and an eosinophilic cationic protein (ECP) over 200 µg/L indicated eosinophilic inflammation.

The bronchoalveolar lavage consisted of 80% eosinophils, with no signs of bacterial infection. The transbronchial lung biopsy indicated eosinophilic pneumonia and no vasculitis (Fig. 1d). There were no clinical or laboratory signs of eosinophilic granulomatous vasculitis or involvement of other organs. After additional testing neither the American College of Rheumatology (ACR) criteria nor Lanham criteria for eosinophilic granulomatosis with polyangiitis were met. Allergic bronchopulmonary aspergillosis was ruled out by low IgE levels and a negative specific IgG to *Aspergillus*.

Given the clinical presentation, laboratory and radiological findings a diagnosis of CIEP was established; however, the patient declined treatment with systemic glucocorticoids. An off-label treatment with mepolizumab was proposed, and after a detailed conversation the patient consented to proceed. The patient was administered 3 monthly doses of 100 mg mepolizumab each. The treatment was discontinued after 3 months due to the symptom-free state and radiological progress.

## Results

**Fig. 2:**
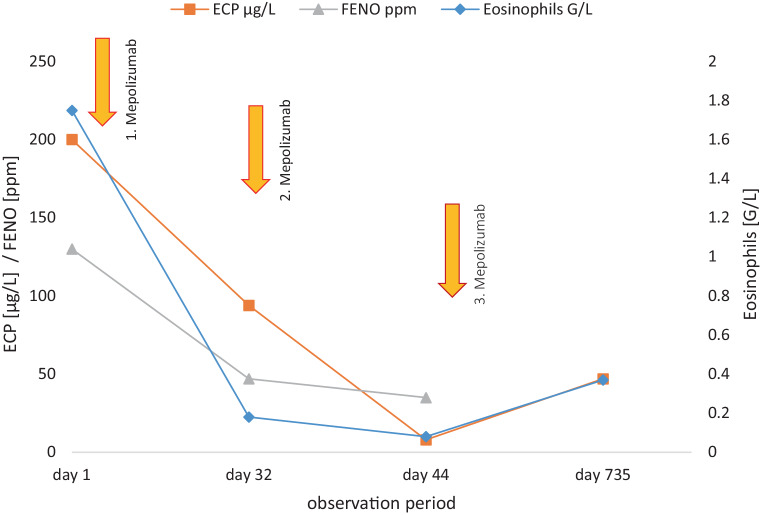
Eosinophilic cationic protein (ECP) in µg/L, fraction of exhaled nitric oxide (FENO) in ppm, and eosinophils in G/L during and after the treatment

The patient experienced full symptom resolution without side effects 2 weeks post-mepolizumab initiation. Radiological follow-up revealed a near-total resolution of the previous consolidations in the chest X‑ray. The chest CT after 9 months (Fig. 1c) confirmed total resolution of the consolidations. Subsequent laboratory tests showed a substantial decrease in FENO and ECP within a few weeks, and peripheral eosinophilia returned to normal levels (Fig. 2). After over 2 years, the patient showed no clinical or radiological recurrence of eosinophilic pneumonia, and the biomarkers consistently remained normal, indicating the sustained efficacy of the treatment.

## Discussion

The CIEP is characterized by eosinophilic lung infiltration [9], manifesting with respiratory symptoms and eosinophilia [10]. Traditionally treated with glucocorticoids, the long-term use is marred by significant side effects and high relapse rates [11].

This case is distinguished by the successful use of mepolizumab in CIEP treatment without glucocorticoids. Mepolizumab’s efficacy in eosinophilic asthma suggests its potential in CIEP management [12]. Our patient showed rapid and sustained improvement with a shorter treatment duration than commonly reported [13]. Considering the infrequent spontaneous resolution of CIEP [14], the rapid and sustained improvement in our patient strongly highlights the efficacy of mepolizumab.

The significant reduction in FENO and ECP levels, established biomarkers in asthma and indicative of airway inflammation, underscores their potential utility in monitoring CIEP [15]. This case suggests their role in tracking disease progression and response to treatment in CIEP, meriting further study.

Our findings underscore the potential of mepolizumab as a glucocorticoid alternative for CIEP, particularly for patients intolerant to steroids. This case bolsters the argument for personalized treatment in CIEP, with a need for broader studies to confirm these results. The effectiveness of FENO and ECP as biomarkers, as evidenced in our case, may transform CIEP management in both research and clinical settings.
